# Quantifying the spatiotemporal dynamics in a chorus frog (*Pseudacris*) hybrid zone over 30 years

**DOI:** 10.1002/ece3.2232

**Published:** 2016-06-26

**Authors:** Kristin N. Engebretsen, Lisa N. Barrow, Eric N. Rittmeyer, Jeremy M. Brown, Emily Moriarty Lemmon

**Affiliations:** ^1^ Department of Biological Science Florida State University 319 Stadium Drive Tallahassee Florida 32306; ^2^ Department of Biological Sciences Museum of Natural Science Louisiana State University 202 Life Sciences Building Baton Rouge Louisiana 70803; ^3^ Research School of Biology The Australian National University Gould Building 116 Canberra ACT 2601 Australia

**Keywords:** Allozyme, hybrid cline, hybridization, microsatellite, Pearl River

## Abstract

Although theory suggests that hybrid zones can move or change structure over time, studies supported by direct empirical evidence for these changes are relatively limited. We present a spatiotemporal genetic study of a hybrid zone between *Pseudacris nigrita* and *P. fouquettei* across the Pearl River between Louisiana and Mississippi. This hybrid zone was initially characterized in 1980 as a narrow and steep “tension zone,” in which hybrid populations were inferior to parentals and were maintained through a balance between selection and dispersal. We reanalyzed historical tissue samples and compared them to samples of recently collected individuals using microsatellites. Clinal analyses indicate that the cline has not shifted in roughly 30 years but has widened significantly. Anthropogenic and natural changes may have affected selective pressure or dispersal, and our results suggest that the zone may no longer best be described as a tension zone. To the best of our knowledge, this study provides the first evidence of significant widening of a hybrid cline but stasis of its center. Continued empirical study of dynamic hybrid zones will provide insight into the forces shaping their structure and the evolutionary potential they possess for the elimination or generation of species.

## Introduction

Hybridization, the ability of individuals from different species to mate and produce viable offspring, has important consequences for the formation and stability of distinct species (Hewitt 1988). Many theoretical and empirical studies of hybrid zones, areas in which contact and hybridization between two species occur, focus on how hybridization is maintained and the long‐term effects of hybridization on either species (Moore 1977; Barton 1979; Barton and Hewitt 1985; Jiggins and Mallet 2000; Mallet 2007; Abbott et al. 2013; Butlin and Ritchie 2013). Hybridization can lead to a variety of outcomes, with potential for a stable hybrid zone, strengthened barriers against gene exchange (reinforcement), collapse of barriers against gene exchange (fusion of species), loss or gain of genetic diversity, or establishment of a novel species (hybrid speciation; Barton and Hewitt 1985; Servedio and Noor 2003; Taylor et al. 2006; Mallet 2007; Abbott et al. 2013). Investigating hybridization in contact zones at different stages of their development is critical to our understanding of speciation and whether individual species barriers will persist or collapse in the face of gene flow (Hewitt 1988; Carney et al. 2000; Buggs 2007; Abbott et al. 2013; Smith et al. 2013a; Beysard and Heckel 2014; Curry and Patten 2014).

Hybrid zones create a natural laboratory in which researchers can study hybridization events and their effects on the speciation process (Hewitt 1988; Harrison 1990; Buggs 2007). The stability and size of a hybrid zone are affected by factors such as individual hybrid fitness, dispersal distance and amount of gene flow with parental species, physical dispersal barriers, and habitat alteration (Moore 1977; Barton and Hewitt 1985; Abbott et al. 2013; Smith et al. 2013a). Many theoretical models have been proposed to explain stable hybrid zone cline structure, which can be roughly sorted into two categories. Dispersal‐independent zones, such as the “bounded hybrid superiority” model (Hagen 1967; Moore 1977), contain hybrids that exhibit a higher fitness in an intermediate environment than their parental types. The size of these zones is primarily regulated by exogenous selection and the geographic extent of the environment that favors the hybrids (Moore 1977; Good et al. 2000). Dispersal‐dependent zones, such as tension zones, consist of hybrids with fitness inferior to parental types (Key 1968; Barton 1979; Barton and Hewitt 1985; Hewitt 1988). Tension zones are maintained by a balance between endogenous selection against hybrids and migration of parental types into the hybrid zone. They are thought to reach equilibrium in areas of low population density but may move if parental ranges shift (Barton and Hewitt 1985; Carling and Zuckerberg 2011; Smith et al. 2013a; Taylor et al. 2015). Theory suggests that clines of either model can move in response to factors such as environmental or climatic change (Hairston et al. 1992; Parmesan et al. 1999; Britch et al. 2001; De La Torre et al. 2015; Taylor et al. 2015), dominance drive (a dominant allele replacing a recessive allele; Mallet 1986), or asymmetrical hybridization (Bronson et al. 2003), but relatively few long‐term studies have successfully and directly documented significant movement in clines of either model (Buggs 2007; Carling and Zuckerberg 2011).

To understand the evolutionary trajectory of hybrid zones, research focused on the structure of hybrid zones must be conducted at multiple time points following secondary contact (Jiggins and Mallet 2000; Carling and Zuckerberg 2011; Beysard and Heckel 2014; Curry and Patten 2014). Many hybrid zone studies, however, are based on a narrow time frame or inconsistent sampling, which provides only a limited view and precludes any inference on hybrid zone stability in evolutionary time. Analogous data spanning two or more sampling periods, although difficult to acquire, are the most direct way to assess a hybrid zone (Buggs 2007; Carling and Zuckerberg 2011; Smith et al. 2013a), and such long‐term studies can reveal complex evolutionary changes in the makeup of the zone (Carney et al. 2000). Some studies have indirectly inferred movement based on the distribution of molecular markers (Rohwer et al. 2001; Gay et al. 2008), but these patterns have been disputed in their ability to successfully identify hybrid zone movement and may instead indicate differential introgression of loci (Barton and Hewitt 1985; Goodman et al. 1999). Buggs (2007; Table 1) documented 23 studies utilizing a variety of data types to show hybrid zone movement, although few of these studies used genetic sampling or covered a significant length of sampling time consistently. Of the handful of genetically based studies that have evaluated cline structure and movement, in only two studies did sampling exceed two decades in length (Carling and Zuckerberg 2011; Smith et al. 2013b; but see also Britch et al. 2001; Dasmahapatra et al. 2002; Taylor et al. 2006, 2014; and review in Buggs 2007). Although these studies provided some evidence for movement of hybrid zones, more research is needed that evaluates how cline structure can change through time. We seek to better understand hybrid zone dynamics by investigating such a zone using sampling of the same genetic markers over three decades.

**Table 1:** Detailed sample collection and population designation. “Collection site” identifies original collection designation of recent samples before pooling. “# from Collection site” gives the number of individuals from that collection site, where “Population sample size (*N*)” gives the number of individuals in each recent population as used in all analyses. “Weighted Latitude/Longitude” for each recent population is a weighted average of the coordinates from the “Collection latitude/longitude” of the 30 recent collections sites that were combined. “Distance along linear transect” indicates position of each population along the transect as calculated for clinal analyses. Collection Site M22 (*n* = 1) was discarded because it was not geographically close enough to combine with any other collections sites during pooling. In analyses, populations A and H1 were designated as pure parental *P. fouquettei*, and populations M, N, O, P, and H7 were designated as *P. nigrita*

Population ID	Collection site	Collection year	# from collection site	Population sample size (*N*)	Collection latitude	Collection longitude	Weighted latitude	Weighted longitude	Distance along linear transect
Recent sampling (West to East across SE US)									
Allopatric *Pseudacris fouquettei*									
A	M1	2006	7	7	30.3309	−91.6964	30.3309	−91.6964	62.242
Putative hybrids of *P. fouquettei* and *P. nigrita*									
B	M23	2001	2		30.68889	−90.88944			
B	M14	2010	9	11	30.70778	−90.88111	30.70434	−90.88263	139.325
C	M16	2010	5		30.775	−90.75917			
C	M15	2010	3	8	30.77444	−90.73333	30.77479	−90.74948	151.918
D	M17	2010	4	4	30.82229	−90.67302	30.82229	−90.67302	159.133
E	M25	2012	5		30.40008	−89.95806			
E	M24	2012	1	6	30.36677	−89.94389	30.39453	−89.9557	228.813
F	M26	2012	5	5	30.40007	−89.90917	30.40007	−89.90917	233.257
–	M22	2003	1	–	30.56551	−89.87149			
G	M5	2003	10		30.384	−89.7554			
G	M2	2003	5		30.35981	−89.75119			
G	M3	2006	3		30.36296	−89.74986			
G	M4	2006	5	23	30.3758	−89.7483	30.37421	−89.75222	248.348
H	M13	2003	1		30.47576	−89.69263			
H	M12	2006	2		30.46701	−89.68592			
H	M20	2012	1	4	30.5102	−89.68387	30.47999	−89.68708	254.347
I	M11	2003	3	3	30.43992	−89.65759			
I	M10	2003	6	6	30.42978	−89.64799	30.43316	−89.65119	257.891
J	M18	2012	5		30.42554	−89.60114			
J	M19	2012	5		30.42898	−89.59735			
J	M9	2006	16	26	30.42702	−89.59648	30.42711	−89.59754	263.043
K	M6	2007	4		30.38572	−89.47497			
K	M7	2007	8		30.39767	−89.44804			
K	M8	2007	10	22	30.41585	−89.43307	30.40376	−89.44613	277.598
L	M21	2003	13	13	30.50104	−88.90835	30.50104	−88.90835	328.889
Allopatric *P. nigrita*									
M	M29	2005	5	5	30.48225	−85.95448	30.48225	−85.95448	611.863
N	M28	2005	5	5	30.7371	−85.91129	30.7371	−85.91129	615.434
O	M27	2003	4	4	30.1437	−84.9766	30.1437	−84.9766	706.278
P	M30	2003	9	9	31.23799	−84.50169	31.23799	−84.50169	749.345
Historical sampling (West to East across SE US)									
Allopatric *Pseudacris fouquettei*									
H1	H1	1976	16	16	31.91924	−92.30716	31.91924	−92.30716	0
H2	H2	1976	4	4	30.4318	−89.90839	30.4318	−89.90839	233.259
Putative hybrids of *P. fouquettei* and *P. nigrita*									
H3	H3	1976	17	17	30.37827	−89.76851	30.37827	−89.76851	246.778
H4	H4	1976	25	25	30.40496	−89.69406	30.40496	−89.69406	253.849
H5	H5	1976	6	6	30.43631	−89.65414	30.43631	−89.65414	257.601
Allopatric *P. nigrita*									
H6	H6	1976	30	30	30.52175	−89.59249	30.52175	−89.59249	263.314
H7	H7	1976	19	19	30.57552	−89.21105	30.57552	−89.21105	299.728

**Figure 1:**
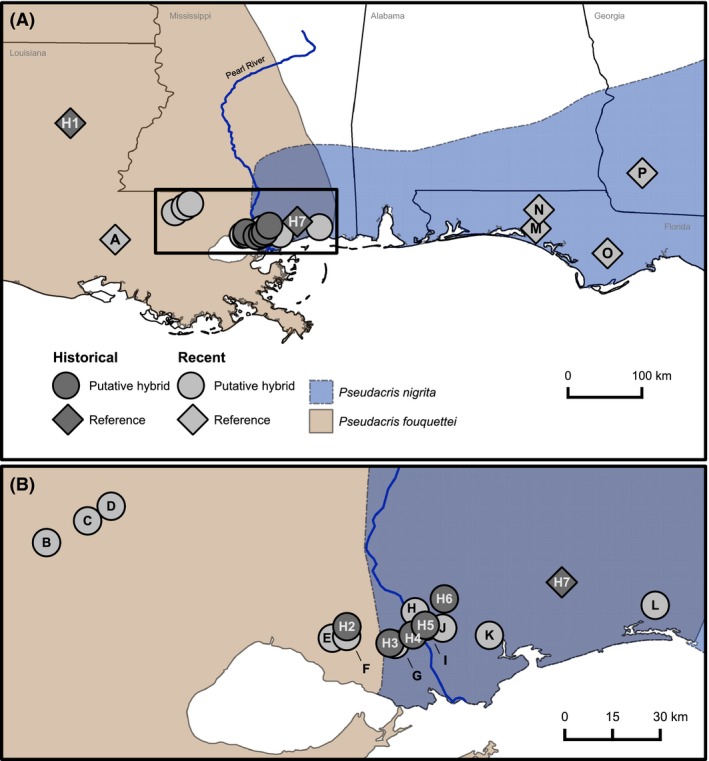
Sampling locations. All 23 populations are shown, with seven historical populations shown in dark gray symbols and 16 recent populations shown in light gray symbols. The range of pure *P. fouquettei* extends to the west and pure *P. nigrita* to the east. Diamonds indicate pure populations used as references for hybrid index analysis, and circles indicate populations tested as putative hybrids. The black box in A corresponds to the enlarged view in B.

The North American trilling chorus frogs (genus *Pseudacris*) are an excellent system to address questions about hybrid zones, because many closely related species in this genus occur in partial sympatry with potential for hybridization, character displacement, or other interactions. There are nine currently recognized *Pseudacris* species belonging to the “Trilling Frog” clade (Moriarty and Cannatella 2004; Lemmon et al. 2007b), and these have been the focus of a variety of studies on speciation (Fouquette 1975; Gartside 1980; Lemmon et al. 2007a, 2007b; Lemmon and Lemmon 2008, 2010; Lemmon 2009). Some *Pseudacris* species have shown evidence of character displacement in advertisement calls and associated female preference when in sympatry with another closely related species (Fouquette 1975; Lemmon 2009). Additionally, in a few regions of species overlap, apparent mitochondrial introgression suggests previous hybridization between closely related species (Lemmon et al. 2007a, 2007b). In one such species pair, recent mitochondrial evidence corroborated allozyme data that described the same hybrid zone previously (Gartside 1980). Although the western species in Gartside's (1980) study was then referred to as *P. triseriata feriarum,* divergent mitochondrial, morphological, and acoustic characteristics from other *Pseudacris* species have since led to its description as a new species, *P. fouquettei* (Lemmon et al. 2007b, 2008). *P. fouquettei* is a congener to *P. nigrita*, differentiated by mtDNA, color pattern, and acoustic signals (Lemmon and Lemmon 2008). Speciation between *P. nigrita* and *P. fouquettei* occurred ~4.8 mya, and their divergence is correlated with marine inundation of the Mississippi Embayment during the late Miocene and early Pliocene, when rising sea levels isolated these taxa geographically (Lemmon et al. 2007a). These two species come together in a narrow contact zone across the Pearl River of southeastern Louisiana and southern Mississippi, where no other species of trilling chorus frogs occur (Fig. 1). Gartside (1980) estimated that the hybrid zone was between 7 and 19 km wide in 1976. He utilized electrophoretic allozyme data from four proteins and gave each individual a hybrid index score based on their genotypes at two markers with fixed differences between the most distant parental populations. Of his seven study localities, three central sites were found to contain hybrid individuals, but no evidence of hybridization was found in either of the two localities to the west (pure *P. fouquettei*) or to the east (pure *P. nigrita*) of the contact zone.

According to Gartside (1980), both breeding between fertile hybrids and backcrossing to parental types were likely occurring to sustain the stable populations of hybrid individuals. The study region has changed significantly since Gartside's sampling in 1976, impacted by both natural disasters and human development. Hurricane Katrina made landfall at the mouth of the Pearl River in 2005, causing high tree mortality and changes in the composition of forest plant species. These changes specifically affected hardwood bottomland forests (Chapman et al. 2008), which is the habitat type Gartside (1980) identified as sustaining hybrid *Pseudacris* populations in the 1970s. In conjunction with the prestorm trend of suburbanization, redevelopment after Katrina led to extensive infrastructure increases in and around the study area. Human and climatic factors could affect both the distribution and population size of the two species in question, and each factor has previously been implicated as a potential driver of change in species distributions (Parmesan et al. 1999; Britch et al. 2001; Taylor et al. 2015).

Acquiring high‐quality historical genetic samples can be problematic, as some methods for storing historical material have been found to make DNA unusable (Taylor et al. 2006). Here, we present successful genotyping and analysis of a historical dataset using tissues collected in the 1970s. We couple this dataset with analysis of recently collected specimens from the study region and analyze the same genetic markers in both datasets to characterize the hybridization between *P. nigrita* and *P. fouquettei* at two points in time roughly 30 years apart. In this way, we have a unique opportunity to directly evaluate temporal changes in the hybrid zone. Our goals for this study are threefold. First, we characterize the genetic diversity in populations of *P. nigrita* and *P. fouquettei* across the Pearl River in both historical and recent times. Second, we compare overall levels of hybridization between time points. Third, we evaluate whether any shift in cline shape or center location has occurred over the past three decades, indicating a change in the balance or strength of forces structuring the cline. Through direct comparison of temporally separated genetic datasets, our study assesses the dynamics of species interactions that create hybrid zones and provides insight into the forces that affect cline stability.

## Materials and Methods

### Historical and recent sampling

For the purpose of this article, samples designated “historical” refer to tissues collected by Gartside (1980). Individuals were sampled between January and March 1976 from seven populations across southern Mississippi and Louisiana. Frogs were euthanized to collect blood, heart and skeletal muscle, and liver tissue. Tissues were stored at −80°C at the Louisiana State University Museum of Natural Science prior to being sent to Florida State University for DNA extraction in 2013. We extracted DNA from 117 samples of adult frogs representing all seven of Gartside's populations, designated as H1 although H7 (Tables 1 and S1).

Samples labeled “recent” include individuals collected between 2001 and 2012. Recent samples were primarily liver tissue, although a small number of samples (*n* < 5) were either toe clips or heart muscle. Tissues were either frozen in liquid nitrogen at collection or placed in 95% ethanol or tissue buffer and stored at −80°C until DNA extraction. The recent dataset includes 161 frogs from 29 collection sites across the southeastern United States, which we combined into 16 recent populations based on geographic proximity, designated alphabetically as A through P (Tables 1 and S1). Uneven sampling across the study area reflects the biological reality of small chorus frog populations during collection of specimens. Individuals are not naturally distributed evenly across the landscape, as they are restricted to areas with appropriate habitat, and further limited by habitat conversion to residential and agriculture use. However, small sample sizes and uneven sampling in both historical and recent datasets make all downstream analyses conservative in their estimates. Additionally, all analyses and methods used in this study incorporate sampling size and scheme to make estimates more robust against sampling disparity.

Both the historical and recent datasets were divided into three zones for analysis to align with prior historical findings: allopatric *P. nigrita* to the east of the contact zone, allopatric *P. fouquettei* to the west, and putative *P. nigrita/P. fouquettei* hybrids within the contact zone around the Pearl River (Fig. 1). Reference populations were chosen far from areas of putative hybridization and were based on the geographic ranges where each species has been shown to be genetically and morphologically distinct (Lemmon et al. 2008; Fig. 1). To determine whether allopatric reference populations of each species were genetically distinct, we employed Bayesian clustering implemented in the STRUCTURE software (v.2.3.4; Pritchard et al. 2000; Falush et al. 2003, 2007) using the microsatellite loci. We explored population values from *K* = 2 to *K* = 8 under the admixture model with 10 replicates per *K*, a burnin of 50,000 generations and 150,000 additional generations sampled. For other parameters, default settings were used. We used Structure Harvester v. 0.6.94 (Earl and vonHoldt 2012) to summarize the replicates for each value of K in both datasets and calculate mean likelihoods for each K. *CLUMPP* (v. 1.1.2; Jakobsson and Rosenberg 2007) and *distruct* (v. 1.1; Rosenberg 2004), were employed to visualize the results (Figure S1).

### DNA extraction and microsatellite data collection

Genomic DNA was extracted from all tissue samples (*n* = 278) using the E.Z.N.A. Tissue DNA Kit (Omega Bio‐Tek, Norcross, GA) following the manufacturer's protocols. Fifteen unlinked tetra‐ and dinucleotide microsatellite loci, previously identified and tested on *P. nigrita* and congeneric species *P. feriarum*, were chosen for amplification (Lemmon et al. 2011; Michelsohn 2012; Table S2) and grouped into four multiplexes. One microsatellite locus (P_fer_c101070) was discarded prior to analysis due to low amplification success within several populations. Multiplexed PCR reactions were carried out with fluorescently labeled forward primers using the Qiagen Multiplex PCR Kit (Qiagen, Inc. Valencia, CA, USA; Table S2). Each reaction consisted of 2X QIAGEN Multiplex PCR Master Mix, 0.2 *μ*mol/L forward and reverse primers, and 20 ng template diluted genomic DNA in a total reaction volume of 10 *μ*L. PCR reactions were conducted on a Bio‐Rad DNA Engine Tetrad^®^ 2 thermal cycler with the following temperature profile: an initial denaturation at 95°C for 15 min, 30–36 cycles of denaturing at 94°C for 30 sec, annealing between 48 and 56°C for 90 sec, and elongation at 72°C for 60–90 sec, and a final extension step at 60°C for 30 min. Fragment analysis with GeneScan dye size standards (500 ROX or 500 LIZ; Table S2) was performed on an Applied Biosystems 3730 Genetic Analyzer at the Florida State University DNA Sequencing Facility. Fragment lengths were determined, and binning completed in Geneious v. 6.0.4 (Biomatters, Auckland, NZ) with manual confirmation of fragment lengths.

### Population genetic diversity analysis

Individuals from 29 recent collection localities were pooled into 16 populations for analysis, combining all individuals from localities within 4 km (Table S3). Mean dispersal distances of *P. nigrita* and *P. fouquettei* individuals have been estimated to be between 131 and 194 m/generation (given a generation time of 1 year), so 4 km was chosen as our cutoff to avoid combining genetically divergent populations (Lemmon and Lemmon 2008). To ensure that pooling was justified, pairwise *F*_ST_ values were calculated for each collection site pair to test for significant divergence using GenoDive v. 2.0b25 (Meirmans and Van Tienderen 2004; Table S3). For downstream analysis, coordinates used for each recent population represented a weighted average of the true collection site coordinates of all individuals in that population.

Micro‐Checker v. 2.2.3 (Van Oosterhout et al. 2004) was utilized to test for the presence of scoring errors and null alleles. Private alleles within each species were determined using GenAlEx v. 6.5 (Peakall and Smouse 2006, 2012). Departures from Hardy–Weinberg equilibrium (HWE) were tested with GENEPOP v. 4.2 using a Markov chain method (Guo and Thompson 1992; Raymond and Rousset 1995; Rousset 2008) and a table‐wide sequential Bonferroni correction was applied to correct for multiple tests (Rice 1989). GENEPOP was also used to test the assumption of no linkage disequilibrium (LD) across all loci using a *G*‐test (Raymond and Rousset 1995). Summary diversity statistics, including effective number of alleles and observed and expected heterozygosity for historical and recent datasets, were calculated using GenoDive. To account for differences in sample size, allelic richness per population was estimated using the R package diveRsity v. 1.9.73 (Keenan et al. 2013).

### Hybrid indices

A hybrid index score (*h*) can be assigned to each individual in a population of putative hybrids by comparing the individual's genotype to two distinct parental populations. A value of 0 or 1 is indicative of a pure parental individual, and any values between 0 and 1 indicate some degree of shared allele frequencies from each parental type. We used a maximum‐likelihood method (Buerkle 2005) in GenoDive ver. 2.0b23 (Meirmans and Van Tienderen 2004) to estimate an index for each putative hybrid in the historical and recent datasets separately, specifying a reference (*h *=* *0) and an alternative (*h *=* *1) parental population. In the historical dataset, population H1 (Fig. 1) was used as the reference of pure *P. nigrita*, and H7 was the alternative of pure *P. fouquettei*, based on Gartside's (1980) designations. Although Gartside's study indicated that populations H2 and H6 consisted solely of parental types, these populations were included in our hybrid index analysis due to their geographic proximity to the center of sampling area. Assigning hybrid indices to these individuals along with the putative hybrids allowed us to assure that our species classifications of all historical individuals corroborated Gartside's classifications despite using different molecular markers. For the recent dataset, population A was chosen as the pure *P. fouquettei* reference, and pure populations M, N, O, and P were pooled for the pure *P. nigrita* alternative. These populations were chosen as references due to their distances from the edge of the contact zone and their locations completely within each species’ allopatric range (Fig. 1).

Classifying interspecific hybrid classes using cross‐specific microsatellites can be difficult, due to variation in allele frequencies present in individual hybrids, as well as to variation in allele frequency estimates of parental populations (Buerkle 2005; Fitzpatrick 2012; Thielsh et al. 2012). A recent study that genotyped laboratory‐created F1 hybrids between *P. nigrita* and *P. feriarum* (a congeneric species to *P. fouquettei* and *P. nigrita*) found that F1 hybrids displayed *h* between 0.5 and 0.75 using the Buerkle (2005) method (Lemmon and Juenger, unpubl. data). Extending this finding to our study, we used the hybrid index boundaries of 0.25–0.75 to classify a hybrid individual from a pure individual across all populations. We also employed a second method to classify individuals as hybrids using the 95% confidence interval (CI) associated with *h* determined in GenoDive. If the CI did not extend to either 0 or 1 (where 0 and 1 indicate the parental populations), the individual is classified as a hybrid of undetermined class. These approaches were shown to successfully distinguish known hybrids from parental individuals by E. M. Lemmon and T. Juenger (unpubl. data), so we employ both methods here.

### Geographic clinal analyses

To fit geographic clines (Szymura and Barton 1986) to the hybrid indices and test for differences in cline parameters between the recent and historical data, we used the hzar package v. 0.2‐5 (Derryberry et al. 2014) in R v. 3.1.1 (R Core Team 2014), which incorporates per population sample size into all models. All population localities were first converted to distances along a one‐dimensional transect spanning the hybrid zone by finding their relative position along a regression line fitted through all (both recent and historical) populations using the linear model (lm) function in R. Geographic clines were then estimated for both the recent and historical data using the mean *h* for each population. In addition, geographic clines were estimated for the recent data excluding populations B, C, and D because these populations had lower hybrid indices than expected given their geographic position. The lack of historical sampling from this specific area precluded testing whether this pattern is a recent phenomenon.

For each dataset (recent, recent without B–D, and historical), we used the Akaike information criterion (AIC; Akaike 1974) to test among five cline models: (1) no tails, (2) an eastern tail, (3) a western tail, (4) symmetrical eastern and western tails, and (5) asymmetrical eastern and western tails. The tailed cline models allow modeling of a “stepped” pattern of steep change near the center of the cline with a more gradual shift in allele frequencies further from the cline center (Szymura and Barton 1986). This “stepped” pattern is frequently observed in empirical data and is thought to be a result of strong linkage disequilibria near the center of the cline, and a resultant rapid shift due to the effects of selection at multiple loci. Further from the cline center, these disequilibria decay through the effects of recombination, and the resultant weaker selection against hybridization allows for the introgression of a few neutral or weakly selected alleles across the species boundary (Szymura and Barton 1986). These tails may also be modeled independently on each side of the hybrid zone to account for asymmetries that may be due to processes such as differential migration, introgression, or selection against hybridization between parental species. Each tail consists of two parameters, *τ* (tau) and *δ* (delta), that describe the shape of decay. These parameters were estimated for each side of the cline individually in the absence of a tail on the other side of the cline (i.e., the eastern and western tail models), constrained to a single *τ* and a single *δ* parameter for both sides of the cline (i.e., symmetrical model), or were estimated independently for each side of the cline (i.e., asymmetrical model). For all analyses, *p*_min_ and *p*_max_, the character values for the pure parental populations, were set to 0 and 1, respectively, in congruence with the *h* values for the pure reference and pure alternative populations. We first estimated covariances between parameters by running a Markov chain Monte Carlo (MCMC) analysis under each model for 10^6^ generations and sampling every 100 generations, following a burn‐in period of 10^5^ generations. Estimated covariances were used to improve MCMC proposals (Derryberry et al. 2014; Gowen et al. 2014). We then estimated cline parameters with three independent MCMC analyses of 10^6^ generations, sampling every 100 generations following a burn‐in period of 10^5^ generations. We assessed convergence by examining MCMC traces for stationarity.

To test for significant differences in cline parameters between the historical and recent sampling, we repeated the cline analyses but constrained the cline width, center, or both from one dataset to the 95% credibility interval estimated for the other dataset. We did this both by constraining the recent to the historical dataset and, reciprocally, constraining the historical to the recent dataset. The historical dataset was analyzed both under the simplest model (i.e., no tails) and the best‐fit model based on the AIC values (i.e., western tail; see Table 2). In this test, parameters were constrained to the estimates from the recent dataset both including and excluding populations B, C, and D. Recent datasets were analyzed under the simplest model (i.e., no tails), which was also the best‐fit model based on AIC values (Table 2), and were constrained to the parameter estimates from the historical dataset analyzed under both its respective simplest and best‐fit models. Constrained analyses were compared to the corresponding unconstrained analyses via likelihood ratio tests and AIC scores.

**Table 2:** Information theoretical statistics for geographic cline model testing. “Model” identifies the multiple models under which the data were analyzed, where the model in bold received the highest weight for that set of models. *E*_min,*i*_ is the evidence ratio, the ratio of the AIC weight of the best‐fit model to that of the model under consideration, or how much less likely each considered model is compared to the best‐fit model

Model	Log likelihood	*k*	AIC	∆AIC	Relative likelihood	Weight	*E*_min,*i*_
Historical sampling							
No tails	−3.8951	2	11.7903	2.9853	0.2248	0.1349	4.4489
**West tail**	−**0.4025**	**4**	**8.8050**	**0.0000**	**1.0000**	**0.6003**	**1.0000**
East tail	−3.8951	4	15.7903	6.9853	0.0304	0.0183	32.8731
Symmetrical tails	−1.8687	4	11.7374	2.9324	0.2308	0.1386	4.3327
Two tails	−0.1183	6	12.2366	3.4316	0.1798	0.1080	5.5612
Recent sampling							
**No tails**	−**4.7955**	**2**	**13.5909**	**0.0000**	**1.0000**	**0.7021**	**1.0000**
West tail	−4.7955	4	17.5911	4.0001	0.1353	0.0950	7.3895
East tail	−4.7955	4	17.5910	4.0001	0.1353	0.0950	7.3893
Symmetrical tails	−4.7955	4	17.5909	4.0000	0.1353	0.0950	7.3891
Two tails	−4.7956	6	21.5911	8.0002	0.0183	0.0129	54.6031
Recent sampling (exc. Pops B–D)							
**No tails**	−**2.5206**	**2**	**9.0412**	**0.0000**	**1.0000**	**0.6106**	**1.0000**
West tail	−2.5206	4	13.0413	4.0000	0.1353	0.0826	7.3892
East tail	−1.6163	4	11.2326	2.1913	0.3343	0.2041	2.9912
Symmetrical tails	−2.5206	4	13.0412	4.0000	0.1353	0.0826	7.3889
Two tails	−1.9397	6	15.8794	6.8381	0.0327	0.0200	30.5410

Although these reciprocal constraint analyses can provide evidence of significant differences in parameter estimates among datasets, elucidating their cause is more difficult. Differences in parameter estimates could be due to differences in sampling between recent and historical datasets or due to actual changes in the hybrid zone. Therefore, to test for the effect of sampling strategy on the cline, a stratified subsampling approach was applied to minimize the differences in sampling strategy between the recent and historical data. Recent populations were grouped by the most geographically proximate historical population: H1 = A, H2 = E or F, H3 = G, H4 = H, H5 = I, H6 = J or K (Fig. 1). For the easternmost historical population (H7), we used two approaches: (1) selecting the most geographically proximate recent population (L) or (2) selecting the westernmost pure parental population of *P. nigrita* (M) to encompass the full geographic range of the dataset. We then ran 100 replicate cline analyses, in which we randomly selected one of the recent populations to match each historical population as grouped above. Individuals in each population were randomly subsampled, so both recent and historical datasets had the same sample size, reducing the sample size to seven localities and either 73 (using population L to match H7) or 77 (using population M to match H7) individuals for both recent and historical data. Distances between populations were similar between historical and subsampled recent data (within a few kilometers). For each replicate, we then re‐estimated the geographic cline using the subsampled data under the simplest model (no tails) for both the recent and historical data. Parameter estimates were considered significantly different if there was no overlap in the 95% credibility interval between the recent and historical data. This approach of using no overlap in 95% credibility intervals should be conservative and increase our confidence that any significant differences are due to shifts in the cline shape or position over time.

## Results

### Microsatellites and genetic diversity

Results of the STRUCTURE analyses clearly indicated that *P. fouquettei* and *P. nigrita* fall into two separate clusters under the most highly supported models, based on the method of Evanno as implemented in Structure Harvester (Evanno et al. 2005; Earl and vonHoldt 2012). This method indicates that *K* = 2 is the most highly supported model for historical microsatellite data and *K* = 3 for recent (Figure S1). These analyses confirm that these species are indeed genetically distinct in their allopatric ranges in both time periods and confirm our sampling strategy of parental and putative hybrid individuals.

Calculation of pairwise *F*_ST_ values for all combined recent collection sites indicated no significant differences between collection sites pooled in the same population, after a sequential Bonferroni correction for multiple tests (Rice 1989), thereby supporting our pooling strategy (Table S3). This pooling scheme resulted in 16 recent populations that were used in all subsequent analyses. In nine of 14 loci, null alleles were detected in fewer than half of the historical and recent populations, and two loci (P_fer_lrc575 and P_fer_DJURT) did not contain any evidence of null alleles. Two additional loci (A_C08d and P_fer_A7NK3_2) contained null alleles in more than half the tested populations and were excluded from further analyses. An additional locus (P_fer_G79VC) was also removed due to a large amount of missing data across both datasets.

The remaining 11 microsatellite loci were characterized in the 7 historical populations and 16 recent populations separately (Table 3). The average number of alleles per locus across all populations was 22.273 (range: 10–33) in the historical dataset and 26.455 (range: 13–39) in the recent dataset. Allelic richness per population, taking sample size into account, on average decreased slightly from historical (AR = 4.096) to recent (AR = 3.712) sampling (Table 3). The number of alleles per population and other summary statistics are provided in Table 3. In the historical dataset, 245 alleles were documented, with 92 alleles (range: 3–12 per locus) private to *P. fouquettei* and 47 alleles (range: 2–20 per locus) private to *P. nigrita*. In the recent dataset, 291 alleles were recorded, with 45 alleles (range: 2–6 per locus) private to *P. fouquettei* and 102 (range: 3–21 per locus) to *P. nigrita*. The historical dataset, considering both pure and hybrid populations, contains 33 alleles not represented in the recent sampling, and the recent dataset includes 79 alleles not represented in historical sampling. These allelic results may be constrained by the small sample size of the *P. fouquettei* recent reference population, so we interpret them with caution. In nearly all populations across both time periods, observed heterozygosity was lower than expected heterozygosity, and *G*_IS_ values (inbreeding coefficient, analogous to *F*_IS_) were positive (Table 3). Average observed heterozygosity decreased slightly from historical sampling to recent, and expected heterozygosity increased slightly (Table 3).

**Table 3:** Genetic diversity between historical and recent populations. Summary diversity statistics across all loci are given by each population, including observed heterozygosity (*H*_o_), expected heterozygosity (*H*_e_), and inbreeding coefficient (*G*_IS_). Allelic richness (AR) is corrected for sample size. Each asterisk in “Out of HWE” indicates one locus–populations pair that significantly deviated from HWE following sequential Bonferroni correction (of 77 historical pairs and 176 recent pairs). The mean *h* (hybrid index score) is an average of *h* for all individuals in the population, where a value of 1 indicates pure *P. fouquettei* and 0 indicates pure *P. nigrita*

Pop ID	*N*	Num alleles	Eff Num alleles	AR	Obs Het (*H*_o_)	Exp Het (*H*_e_)	Inbreed coeff (*G*_IS_)	Out of HWE	Mean *h*
Historical populations									
H1	16	13.091	8.907	5.12	0.741	0.854	0.132	*	1
H2	4	4.727	3.671	3.61	0.727	0.799	0.09	**	0.911
H3	17	8.818	5.313	4.14	0.678	0.809	0.162	*	0.893
H4	25	10.273	6.108	4.39	0.684	0.791	0.135		0.824
H5	6	4.909	3.414	3.55	0.773	0.75	−0.03	*	0.756
H6	30	8.364	4.397	3.8	0.68	0.727	0.064		0.087
H7	19	9	5.381	4.06	0.697	0.727	0.042		0
Recent populations									
A	7	7.182	5.483	4.27	0.764	0.845	0.096		1
B	11	9.273	6.855	4.62	0.729	0.865	0.157	*	0.604
C	8	8.273	6.394	4.35	0.602	0.851	0.292	*	0.599
D	4	5	3.938	3.7	0.614	0.871	0.296		0.547
E	6	5	3.957	3.58	0.644	0.794	0.189		0.698
F	5	5.091	3.988	3.62	0.623	0.801	0.222		0.66
G	23	9.818	5.345	4.1	0.613	0.77	0.204	**	0.563
H	4	4.545	3.697	3.55	0.682	0.788	0.135		0.595
I	9	6.727	4.631	4.01	0.688	0.813	0.154	*	0.511
J	26	9.909	5.978	4.14	0.678	0.748	0.093		0.279
K	22	11	6.087	4.27	0.609	0.806	0.244	****	0.323
L	13	5.727	3.856	2.83	0.618	0.76	0.187	*	0.276
M	5	5	3.732	2.56	0.613	0.794	0.228		0
N	5	4.375	3.381	2.43	0.65	0.75	0.133		0
O	4	5	4.308	2.7	0.75	0.875	0.143		0
P	9	8.636	6.159	4.66	0.797	0.87	0.084	**	0

Some population–locus pairs showed departures from HWE following sequential Bonferroni correction for multiple tests, but patterns were not consistent across all loci (Table 3). Each population but one displayed a positive *G*_IS_ value, indicating a deficiency in the number of heterozygotes captured in the sample compared to HWE expectation. In order to determine that slight departures from HWE did not affect our determination of hybrid status, we removed all population/locus pairs that were out of HWE in either dataset and recalculated hybrid indices. As the correlation between the original and recalculated *h* scores was strong (*r* ^2^ = 0.958), the original *h* values and hybrid indices were used with confidence in all downstream analysis. No evidence of LD was detected across loci in either the historical or recent dataset following standard Bonferroni correction.

### Hybrid indices

**Figure 2:**
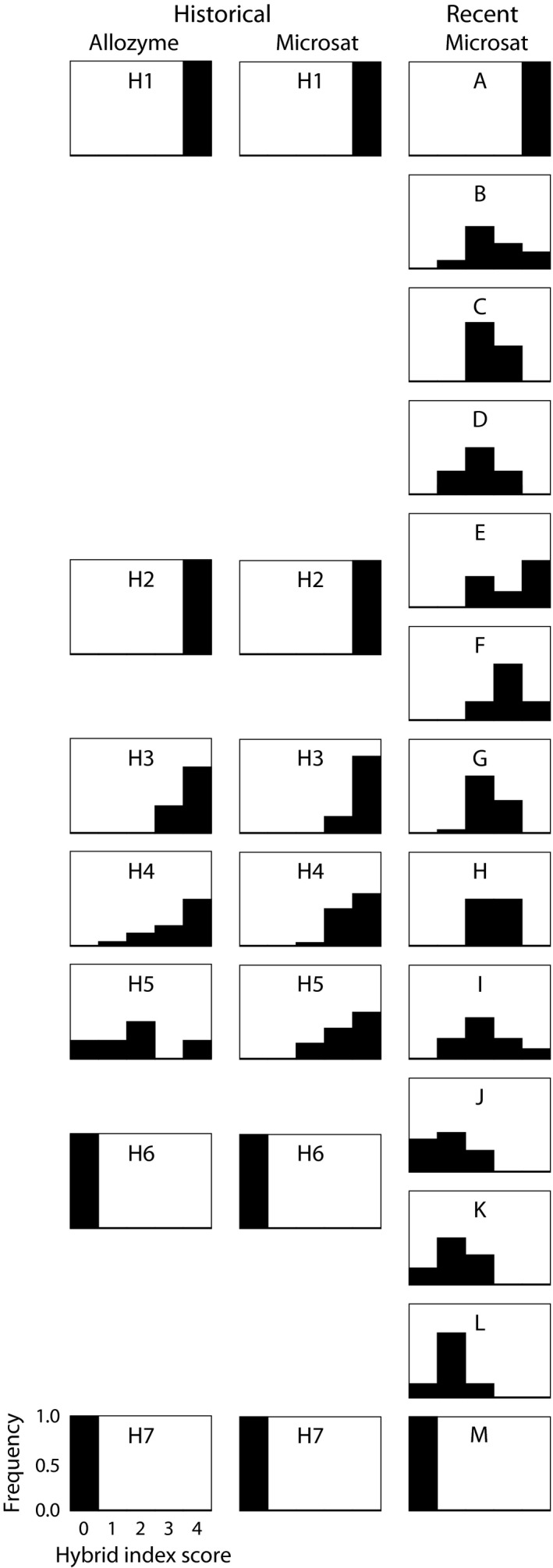
Distribution of hybrids. The frequency and proportion of hybrid and pure individuals in each population are represented by their hybrid index score (*h*) where 0 equals pure *P. nigrita* and 4 equals pure *P. fouquettei*. The historical allozyme data are from Gartside (1980, Fig. 3). Historical and recent populations are matched as they were for subsampling analyses. Hybrid index scores correspond to this scale: 0 = 0.0 to 0.2; 1 = 0.2 to 0.4; 2 = 0.4 to 0.6; 3 = 0.6 to 0.8; 4 = 0.8 to 1.0.

Hybrid index scores for all individuals were compared to assess the frequency and distribution of hybrids across the historical and recent datasets, including current and 1980 data (Fig. 2). *h* scores for putative hybrids were graphed from west to east to visualize geographic structure across the hybrid zone (Figure S2). Based on our microsatellite analysis of the historical dataset, no individuals in populations H2 or H6 were assigned a hybrid index between 0.25 and 0.75. This agrees with Gartside's 1980 results that these populations contained only parental types (Fig. 2; Table S1). Also consistent with Gartside's (1980) result, we documented hybrids in populations H3, H4, and H5 in the historical dataset, with proportions of hybrids equal to 0.12, 0.28, and 0.5, respectively, using the 25%/75% cutoffs for *h* with microsatellite data. Gartside (1980) originally estimated hybrid individuals from these three populations as 0.29, 0.48, and 0.6, respectively, using allozyme data (Gartside 1980). Differences in these proportions may be due to both resolution of the genetic marker and variation in the sensitivity of the statistical method to identify hybrids. In the recent dataset, each putative hybrid population (B through L) was made up of at least 50% hybrid individuals based on 25%/75% cutoffs for *h*, and 73.28% of all individuals within these 11 populations were classified as hybrids. Using the CI method, there were many more individuals classified as hybrids in both the historical and recent dataset based on the failure of the CI to include either 0 or 1 (Figure S2). In the historical dataset, 69.5% of individuals from populations H2 through H6 were classified as hybrids with the CI method, including individuals from both H2 and H6. Likewise, this method classified 93.1% of individuals from recent populations B through L as hybrids. Although the CI method may be overly liberal in classifying hybrids, both methods are consistent in suggesting that hybrids are much more common in the recent than in the historical dataset. This result suggests that hybridization has increased and expanded past the original boundary described by Gartside (1980).

We also used our STRUCTURE results to examine mixed ancestry of individuals and attempt to identify hybrid individuals. The historical data generated a best‐supported plot (*K* = 2) with cleanly defined clusters, where putative hybrids clustered with one or the other parental species (Figure S1; Evanno et al. 2005). The recent data generated plots (best supported is *K* = 3) with more uncertain placement across all populations, which could be indicative of increased gene flow across the range (Figure S1). To assess whether STRUCTURE Q‐scores (probability of belonging to one cluster vs. another) are comparable to *h* scores from hybrid analysis, we examined these values for known historical hybrids from Gartside's (1980) study. We observed that STRUCTURE does not effectively identify mixed ancestry using microsatellites, suggesting that methods developed specifically for estimating hybrid index, such as those employed above, are more accurate for identifying mixed ancestry and hybridization than cluster‐based methods.

### Clinal analyses

To ensure the hzar MCMC analyses were sampled well enough to yield accurate estimates of the true maximum likelihood, we examined the posterior samples across the different models and datasets. Plots of the posterior distributions show dense sampling around the maximum likelihood, indicating that the maximum likelihood value estimated from the MCMC is sufficiently close to the true maximum likelihood so as to have no impact on the model testing results (Figure S3).

**Figure 3:**
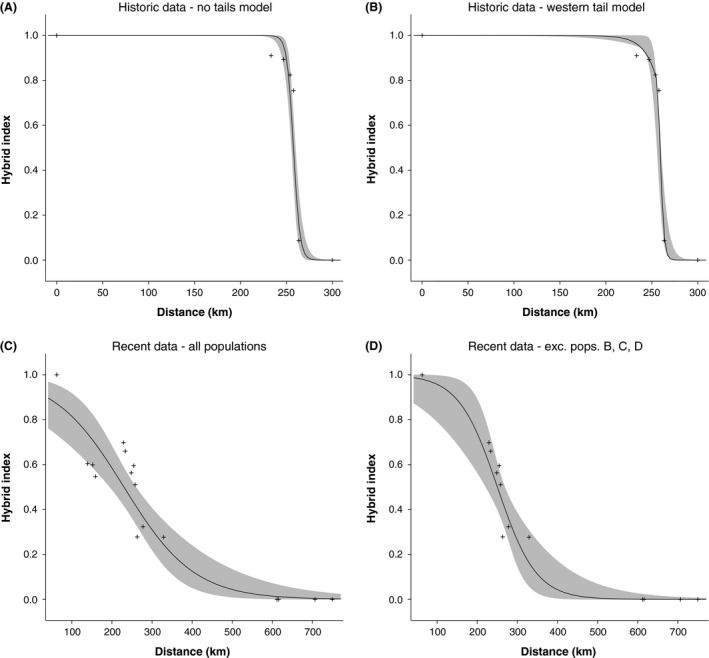
Geographic clines for historical and recent datasets. Panel A depicts the no‐tails model for historical sampling, and B shows the west‐tails model, which was the best‐supported model for the historical sampling. Panels C and D depict the no‐tails models of the recent dataset, where C includes all populations, and D excludes populations B, C, and D.

Geographic cline models including only a western tail were the best fit to the historical data, while models including no tails were the best fit to the recent data, regardless of whether populations B, C, and D were included in the analysis (Table 2, Fig. 3). However, the maximum AIC weights were relatively low (<0.75) across all datasets, and alternative models cannot be rejected (Table 2). Estimates of the cline center were similar across datasets, and credibility intervals broadly overlapped (Table 4). However, the clines widths were substantially larger for the recent datasets (from approximately 10 km historically to 200–300 km recently), particularly when including populations B–D (Table 4).

**Table 4:** Cline parameters. Geographic cline center and width estimates (in kilometers) for the two‐parameter, no‐tails model and, in the case of the historical sampling, the best‐fit model (four‐parameter west‐tail model). The range of values in the 95% credibility interval for each estimate is given in parentheses

Model	Center	Width
Historical sampling		
No tails	257.532 (255.620–259.487)	13.977 (9.898–21.074)
West tail	258.789 (255.619–259.487)	8.654 (8.653–21.071)
Recent sampling		
No tails	230.186 (195.238–259.191)	349.830 (232.372–582.217)
Recent sampling (exc. Pops B–D)		
No tails	239.819 (221.395–268.026)	193.162 (109.162–396.677)

Reciprocal constraints further support (1) the stability of the cline position over time and (2) a significant increase in the width of the hybrid zone from the historical to recent dataset. Analyses in which only the cline center was constrained were not significantly worse than unconstrained analyses across any tests (Table 5). Analyses in which the cline width was constrained, however, were significantly worse than unconstrained analyses (2LLR < −44.837; Table 5), and likelihood ratios were similar between analyses in which only the cline width was constrained and those in which both the cline width and center were constrained. Comparisons using AIC scores corroborate these results: differences in AIC scores were slight (<5.143; Table 5) when only cline centers were constrained, but constrained analyses were consistently significantly worse when the cline width was constrained (∆AIC > 40.837; Table 5).

**Table 5:** Likelihoods and full model testing statistics for reciprocally constrained analyses. “Model” refers to the model under which the data were analyzed, while “Constraint” refers to the model from which the parameter constraints stem. “2LLR” refers to the likelihood ratio (2 × (ln Lcon. − ln Luncon.) comparing the unconstrained likelihood to that obtained by restricting the cline center, cline width, or both. “∆AIC” refers to the difference in AIC between the unconstrained and constrained models (i.e., AIC_uncon_. − AIC_con_.)

Model	Constraint	Uncon. likelihood	Uncon. AIC	Con. Like. Center	Con. AIC Center	2LLR Center	∆AIC Center	Con. Like. Width	Con. AIC Width	2LLR Width	∆AIC Width	Con. Like. Both	Con. AIC Both	2LLR Both	∆AIC Both
Historical sampling															
No tails	Recent	−3.895	11.790	−3.895	9.790	0.000	2.000	−30.220	62.440	−52.650	−50.650	−30.220	60.440	−52.650	−48.650
No tails	Recent (exc. B–D)	−3.895	11.790	−2.323	6.647	3.143	5.143	−30.223	62.445	−52.655	−50.655	−30.220	60.440	−52.650	−48.650
West tail	Recent	−0.403	8.805	−3.895	13.790	−6.985	−4.985	−22.820	51.639	−44.834	−42.834	−22.821	49.642	−44.837	−40.837
West tail	Recent (exc. B–D)	−0.403	8.805	−1.646	9.292	−2.487	−0.487	−22.822	51.644	−44.839	−42.839	−22.821	49.642	−44.837	−40.837
Recent sampling															
No tails	Gartside (No Tails)	−4.796	13.591	−6.089	14.177	−2.586	−0.586	−248.311	498.621	−487.030	−485.030	−249.220	498.440	−488.849	−484.849
No tails	Gartside (West Tail)	−4.796	13.591	−6.088	14.176	−2.585	−0.585	−248.352	498.705	−487.114	−485.114	−249.254	498.509	−488.918	−484.918
Recent sampling (exc. Pops B–D)															
No tails	Gartside (No Tails)	−2.521	9.041	−2.695	7.390	−0.349	1.651	−71.149	144.297	−137.256	−135.256	−71.148	142.296	−137.255	−133.255
No tails	Gartside (West Tail)	−2.521	9.041	−2.695	7.389	−0.348	1.652	−71.162	144.325	−137.283	−135.283	−71.161	142.322	−137.281	−133.281

**Figure 4:**
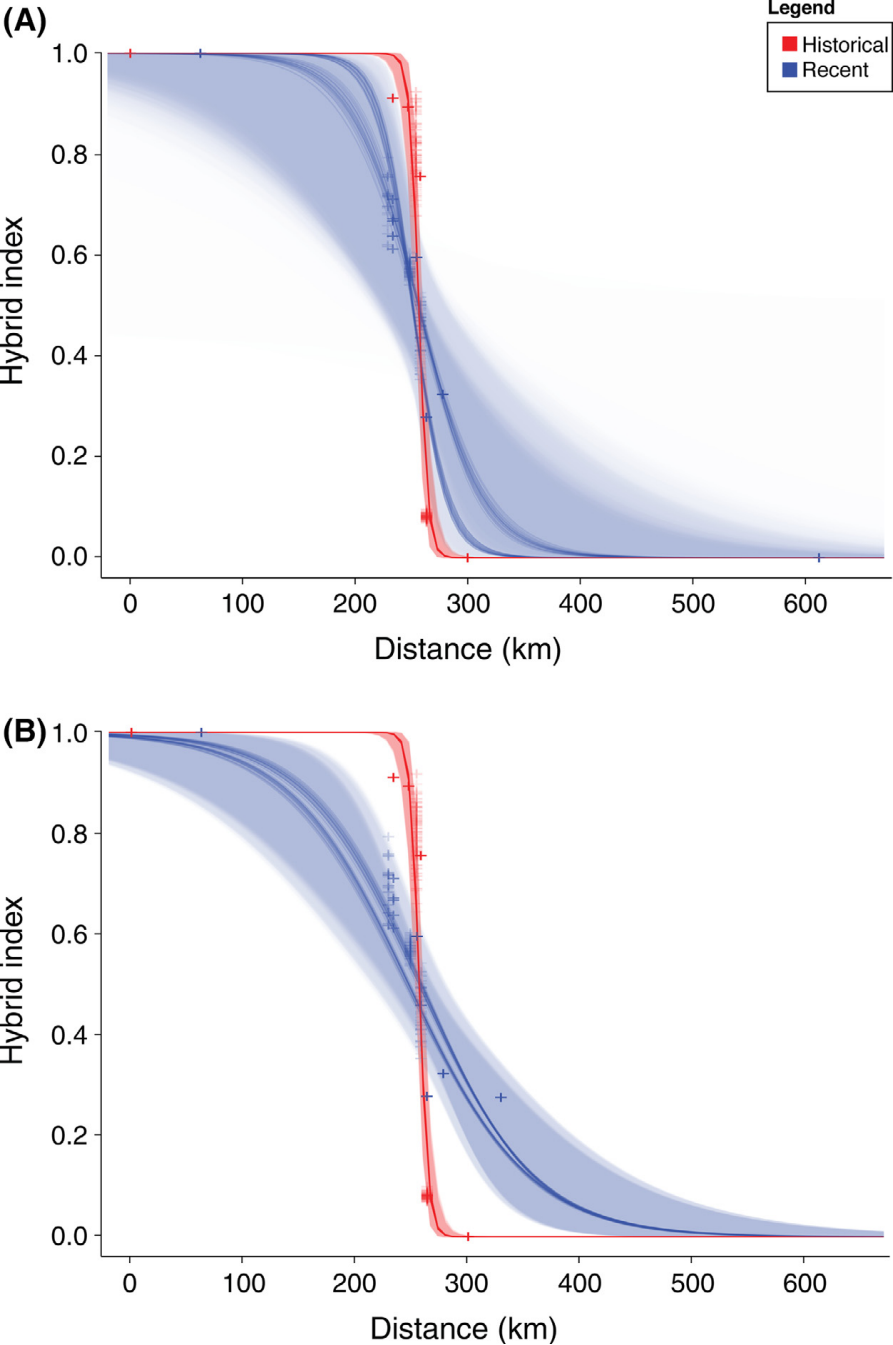
Stratified subsampling clines. Each historical population was matched to a recent population using the stratified subsampling approach to create equal sample sizes, then geographic clines were re‐estimated under the simplest model (no tails). Panel A uses population L as the easternmost recent population, and Panel B uses M as such. Clines are overlaid where red lines indicate historical populations and blue lines indicate recent populations.

Stratified subsampling analyses were highly consistent across replicates in both historical and recent datasets (Fig. 4). However, individuals from recent population J had, on average, lower hybrid indices than individuals from population *K* (Table 3), resulting in a slightly narrower and westward‐shifted cline when selected as the geographic equivalent of historical population H6, although these differences were not significant (Fig. 4). Regardless of whether the most geographically proximate recent population (L) or the westernmost pure *P. nigrita* population from Florida (M) was used as the recent equivalent of population H7, stratified subsampling analyses show no evidence of a shift in cline center position (Fig. 4). Credibility intervals in cline center overlapped substantially in all replicates, indicating a lack of any significant shift in cline center position (Table 6). Stratified subsampling results for cline widths similarly corroborated other analyses. When the pure *P. nigrita* population (M) was selected, stratified subsampling analyses supported a significant increase in cline width, with no overlap in credibility intervals between historical and recent datasets in 99% of the replicates (Table 6). When the geographically most proximate population (L) was selected, similar results were obtained and recent cline width was significantly broader in 100% of the replicates (Table 6). The stratified subsampling results of increased cline width but no change in cline center indicate that limited recent sampling did not affect estimates. Increased stochasticity from limited sampling would have also caused wider and more inconsistent estimates in subsampled historical width, which is not shown by our models. Thus, we are confident that changes seen in cline width are caused by true expansion of the hybrid zone and not due to sampling scheme.

**Table 6:** Stratified subsampling estimates. The mean cline center and width estimates across all 100 replicates from the stratified subsampling analyses are given, using either population L or population M as the easternmost recent population. The range of values from 100 replicates is given in parentheses below each mean. The number of replicates in which the recent parameter estimates were significantly different from historical parameters is also indicated (“Recent Sig. East/West” and “Recent Sig. Narrower/Wider”)

	Pop. L	Pop. M
Historical Center	256.505 (256.066−256.505)	256.487 (256.086−256.961)
Recent Center	252.506 (243.796−260.552)	253.810 (250.0237−258.520)
Recent Sig. East	0	0
Recent Sig. West	0	0
Historical Width	16.311 (15.591−17.231)	16.288 (15.580−17.075)
Recent Width	208.546 (190.005−224.979)	91.565 (54.782−135.447)
Recent Sig. Narrower	0	0
Recent Sig. Wider	100	99

## Discussion

### Expansion of the hybrid zone over 30 years

Movement of hybrid zones, although theoretically supported, has been documented in relatively few conclusive long‐term empirical studies. Of these select studies, only a small number have used analogous genetic data from multiple time points to assess spatiotemporal dynamics of a hybrid zone. Using equivalent genetic sampling of the hybrid zone at two points separated by roughly 30 years, we observed a significant increase in the width of the cline between *P. fouquettei* and *P. nigrita* populations, from 8.7–14 km to 349.8 km, and also confirmed stasis in the center position of the cline. To the best of our knowledge, this widening of the hybrid zone, but stasis of its center, makes our system unique among previous studies of hybrid zone movement. Three studies utilizing between 10 and 20 years of genetic and morphological sampling in crickets, butterflies, and chickadees all documented significant movement in the center of the hybrid zone, but no change in width (Britch et al. 2001; Dasmahapatra et al. 2002; Taylor et al. 2014). Similarly, a study spanning 40 years in an Australian *Litoria* tree frog hybrid zone found a slight shift in cline center position, but no change in cline width (Smith et al. 2013a). Carling and Zuckerberg (2011) demonstrated a different trend through a 40‐ to 50‐year genetic study of the *Passerina* bunting hybrid zone by documenting significant narrowing of hybrid cline width through time and a nearly significant shift in center, which suggests a reduction in hybridization across that contact zone.

In contrast to these findings, hybrid index scores in our data clearly indicate that most individuals in the contact area are hybrids, showing substantial increase in the degree of hybridization. This zone's structure matches the definition of a unimodal zone and hybrid swarm, instead of the bimodal structure characteristic for a tension zone (Jiggins and Mallet 2000; Gay et al. 2008), which was the model Gartside (1980) used to describe the historical zone. The geographic extent of hybridization has also increased since first being characterized in the mid‐1970s and now reaches farther into the ranges of both parental species. While patterns of sampling were not identical between the historical and recent datasets, both subsampling and reciprocal constraint analyses corroborate a widening of the zone and strongly suggest that this finding is not simply the result of sampling differences. These results indicate that *P. fouquettei*/*P. nigrita* hybrids are more geographically widespread than historically recognized, alluding to changes in the maintenance and regulation of this hybrid zone, as well as a need to re‐examine which model best describes this zone.

### Potential drivers of cline expansion

Gartside (1980) described the hybrid zone between these species as “relatively steep” and narrow, extending only between 7 and 19 km wide. Although referring to this zone as one of “parapatric hybridization,” or a tension zone restricted by endogenous selection, he also suggested that exogenous selection was restricting the hybrids to a small region dominated by mixed hardwood bottomlands and flanked by pinewoods. He proposed that hybrids may not be “at an absolute disadvantage to parental types” here as they were throughout the rest of the parental range and that this intermediate zone may be stable for an extended time (Gartside 1980).

A change in exogenous selection could be expected to alter dispersal‐independent hybrid zones if the environment that favors hybrids expands and allows hybrids to expand their range (Hairston et al. 1992). For example, Hairston et al. (1992) demonstrate movement of a hybrid cline between salamanders (genus *Plethodon*) and propose a recent selective advantage for traits of one species over the other, driven by human modifications to the environment. In dispersal‐dependent (tension) zones, however, movement may be expected either when shifts occur in parental density and dispersal or when endogenous selection pressure on hybrids changes (Barton and Hewitt 1985; Buggs 2007; Carling and Zuckerberg 2011; Smith et al. 2013b). Unidirectional expansion of one species into the range of the other may indicate superior competitive abilities or more successful reproduction in the first species as they disperse (Gay et al. 2008). However, in the *P. fouquettei/P. nigrita* complex, it appears that neither parental species has a selective advantage, evidenced by increased width of the hybrid zone on both sides into parental ranges. This expansion suggests that the hybrid zone is no longer stable as a tension zone trapped in a narrow region, as theorized originally (Gartside 1980). Either reduced selective pressure (endogenous or exogenous) against hybrids or increased migration of individuals beyond the historical zone is likely responsible for the changes in this hybrid zone over the past few decades.

One possible explanation for the recent expansion in the historically narrow *P. fouquettei/P. nigrita* hybrid zone could be a reduction in endogenous selection against hybrids through relaxation of prezygotic or postzygotic isolating mechanisms. We think this explanation is unlikely as there is no documented support for strong selection or reproductive isolation historically. No evidence for strong prezygotic reproductive barriers acting against hybrids has been noted, as Gartside stated that male signals of the two species are “essentially similar,” and thus, females would not likely be able to discriminate against heterospecific signals (Gartside 1980; Lemmon et al. 2008). Likewise, there has been no evidence for strong postzygotic reproductive barriers against hybrids, as these species have been shown to have a high level of genetic compatibility to produce hybrid offspring (Mecham 1965; Gartside 1980). This cross can produce both fertile F1 hybrids and future generation hybrids, both mating between two F1 individuals and backcrossing to parental types, indicating low endogenous selection against hybrids in the means of hybrid unfitness (Mecham 1965; Gartside 1980). However, further studies on both female discrimination success and hybrid fitness may reveal previously unnoted historical reproductive isolation and help elucidate the current strength of endogenous selection and reproductive isolation against hybrids.

A more likely factor allowing expansion in this system could be reduced exogenous, or environmental, selection on hybrids. Climatic changes and catastrophic weather events may contribute to exogenous selection by affecting range boundaries of parental species, leading to movement of hybrid zones (Britch et al. 2001; Chapman et al. 2008; Taylor et al. 2015). Over the past 30 years, changes across the Pearl River driven by hurricanes and human influences could have caused expansion of a “strip of intermediate or novel habitat” consisting of “mixed hardwood bottomlands” in which Gartside (1980) noted the hybrids were most successful. In their initial description of *P. fouquettei,* Lemmon et al. (2008) found that this species tends to inhabit and tolerate a broader environmental habitat range than its congener*, P. nigrita,* which prefers pine flatwoods (Fouquette 1975). It is possible that hybrid individuals may also be able to tolerate a broader environment than *P. nigrita* and can outcompete them in their parental range. Furthermore, if environmental changes have resulted in the “intermediate” habitat in which hybrids were more successful then parentals becoming broader in the past three decades, hybrids may now be very successful in geographic areas historically dominated by *P. fouquettei*. The widening of the cline in both directions that we have observed since Gartside's sampling may indicate that hybrids can outcompete both parental species in their historical ranges, but ecological selection on hybrids in any habitat type remains to be tested.

Another potential explanation for expansion of the hybrid zone could be an increase in migration and dispersal due to anthropogenic influence in the past 30 years. Natural and artificial boundaries can restrict dispersal, which will also restrict movement of hybrid zones. When Gartside (1980) studied this area, the level of residential development was low and the area was primarily rural (Chamberlain and Bigelow 2001). Development had increased significantly in this region by the time recent individuals were collected. Anthropogenic influence caused by development could affect chorus frog species in two ways. First, the pressures of increased development could drive dispersing individuals farther from natal regions to find suitable habitat. Competition for mates and other resources may be greater in smaller habitat patches, necessitating increased individual dispersal. A second influence could be caused by the construction of roadside ditches. When roads are constructed to serve newly developed areas, drainage ditches are created that can supply breeding habitat and migration corridors for small amphibians. Studies of another tree frog, *Hyla squirella,* found that roadside ditches increased gene flow among populations of this species in urbanized areas (Hether and Hoffman 2012). In addition, many of the specimens in the present study were collected from such roadside ditches throughout the study area. Thus, it is possible that recently constructed ditches across the *P. nigrita/P. fouquettei* hybrid zone allow increased gene flow and dispersal of individuals.

In addition to change from human influence, the environmental changes brought by weather patterns and major climatic events, such as hurricanes, have the potential to increase individual dispersal by altering habitat. Heavy flooding and high winds during Hurricane Katrina likely damaged chorus frog habitat and may have forced individuals to move from highly impacted areas. Rain and flooding have also been implicated in increasing anuran dispersal by allowing macrophyte rafting along and across rivers, which can cause abnormally long‐distance dispersal (Schiesari et al. 2003; Upton et al. 2014). Macrophyte rafts can also serve as suitable temporary habitat during times when terrestrial habitat is flooded, allowing genetic material to be exchanged through the zone more rapidly (Schiesari et al. 2003; Upton et al. 2014).

When Gartside studied this zone in the mid‐1970s, he found a narrow, sharp cline containing hybrids with both genetic and morphological evidence of intermediate characters. Approximately 30 years later, we find a significantly wider cline displaying predominantly hybrid individuals. The current hybrid zone is broad (349.8 km) relative to estimates of per‐generation dispersal distance (approximately 131–194 m/generation, assuming a generation time of 1 year) for *P. nigrita* and *P. fouquettei* (Lemmon and Lemmon 2008). The width of a tension zone is regulated by dispersal and selection (Key 1968); therefore, a very wide cline, as we observe here, necessitates either uncharacteristically far dispersal of parentals or very low selection pressure on hybrids (Barton and Hewitt 1985; Shapiro 1998). However, Barton and Hewitt (1985) also indicate that hybrid zones found to be wider than expected may be explained by underestimated dispersal rates, supporting a hypothesis of increased individual dispersal. Conversely, a broad cline may imply neutral introgression and indicate that the hybrid zone is not regulated by selection against hybrids at all (Hewitt 1988; Shapiro 1998). We cannot disregard the possibility of a neutral cline, characterized by an “initially steep gradient” that gradually weakens but maintains its center at first contact (Barton and Hewitt 1985).

Finally, ongoing fusion or collapse of two species into one cannot be overlooked, due to the apparent lack of strong reproductive barriers or discrimination among mates. Since timing of the initial contact between *P. fouquettei* and *P. nigrita* is unknown, it is possible that this contact zone was relatively young when Gartside first described it in 1980. A young contact zone in this scenario could have two potential conclusions: (1) The species may be moving toward stable equilibrium, as predicted by the tension zone model (Key 1968; Barton 1979; Barton and Hewitt 1985), or (2) the species may be moving toward either total speciation or fusion by introgression, as predicted by the ephemeral‐zone hypothesis (Dobzhansky 1940; Moore 1977). This possibility of species fusion and collapse is similar to the recent apparent collapse of two sympatric stickleback species (Taylor et al. 2006). If the potential collapse is ecologically based, it may be especially accelerated if anthropogenic influences in the Pearl River region continue to expand.

## Conclusion

We have characterized a dynamic hybrid zone between two trilling chorus frogs, *P. fouquettei* and *P. nigrita*, over a 30 year period, including strong evidence for a dramatic increase in its width but stasis of its center. To our knowledge, this study provides the first evidence of this spatiotemporal pattern in a hybrid system and contributes important empirical evidence to the theory and understanding of hybrid zone movement. In the future, we hope to better understand the processes driving these patterns through estimating hybrid fitness via laboratory experiments, testing female discrimination via phonotaxis experiments, and evaluating recent changes in ecosystem conditions. Future time‐stratified sampling and genetic analysis of individuals throughout the contact region in the future will provide a more thorough understanding of the extended stability of this hybrid zone. This study exemplifies the critical need for consistent long‐term field studies to increase understanding of hybridization and speciation, as well as for studies of the anthropogenic and natural influences on these dynamic systems.

## Data Archival Location

Dryad Data Repository (www.datadryad.com).

## Conflict of Interest

None declared.

## Supporting information


**Figure S1.** STRUCTURE Plots.

**Figure S2.** Hybrid Index score comparisons.

**Figure S3.** Posterior distributions from geographic cline analyses.

**Table S1.** Complete listing of specimens.
**Table S2.** Microsatellite marker information.
**Table S3.** *F*_ST_ and *P*‐values for combined collection site pairs.
