# Supplementary figures and images for: Quantifying the spatiotemporal dynamics in a chorus frog (Pseudacris) hybrid zone over 30 years
Source: Ecol Evol. 2016 Jun 26;6(14):5013–31. doi: 10.1002/ece3.2232 (PMC4979724; doi:10.1002/ece3.2232)

## Historical

## Recent

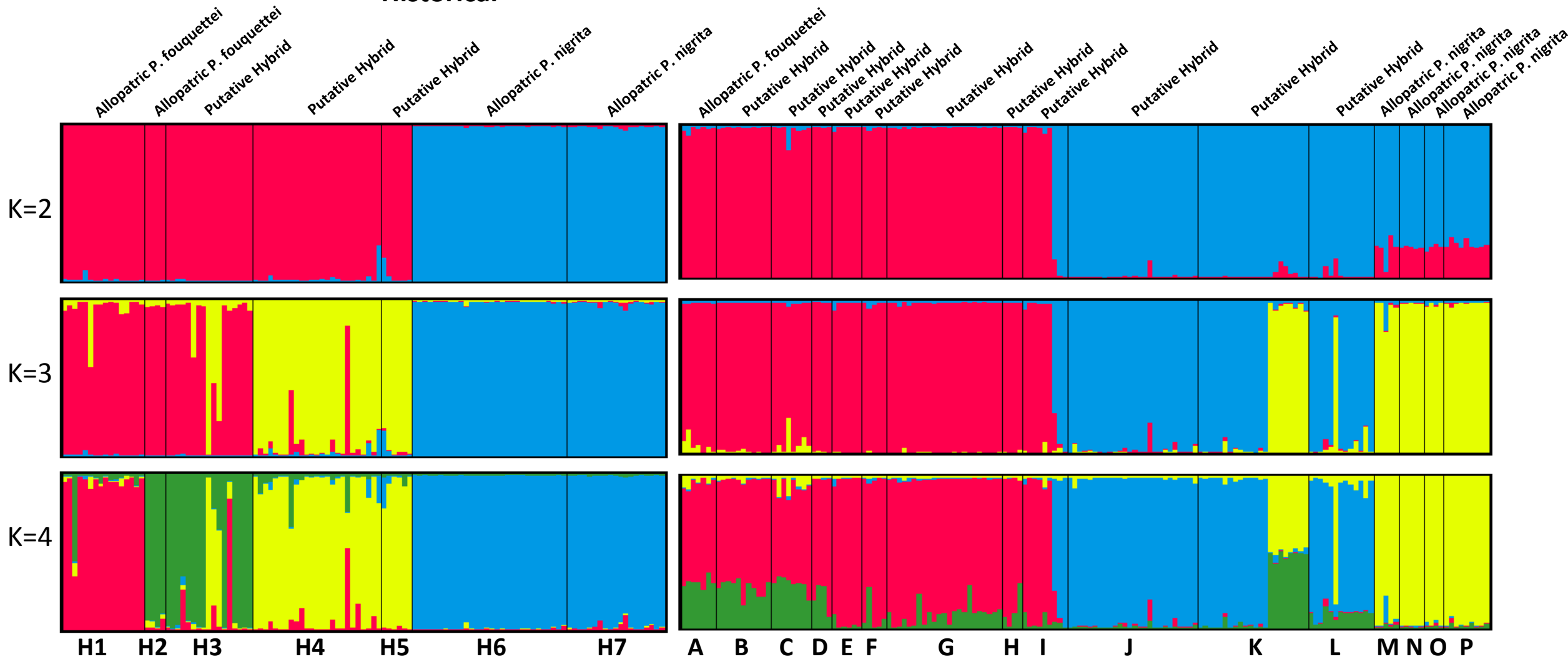

Supplement: Supplementary file 1 — Figure S1. STRUCTURE Plots. [file ECE3-6-5013-s001.pdf]

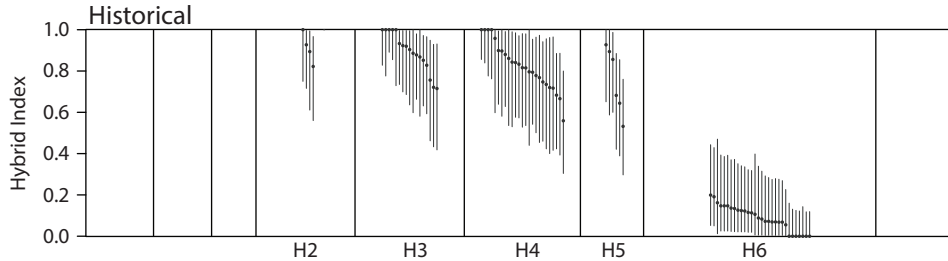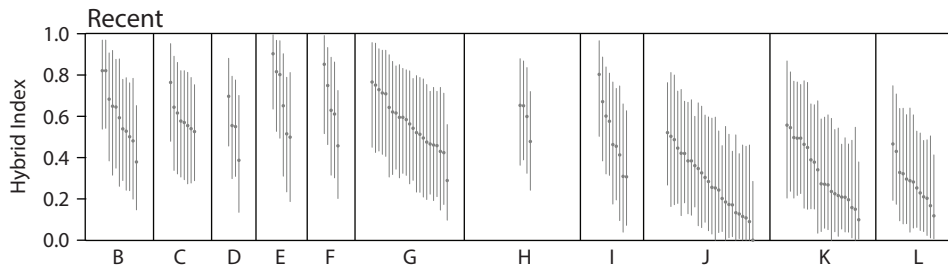

Supplement: Supplementary file 2 — Figure S2. Hybrid Index score comparisons. [file ECE3-6-5013-s002.pdf]
