# Supplementary material for: Quantifying the spatiotemporal dynamics in a chorus frog (Pseudacris) hybrid zone over 30 years
Source: Ecol Evol. 2016 Jun 26;6(14):5013–31. doi: 10.1002/ece3.2232 (PMC4979724; doi:10.1002/ece3.2232)
Supplement: Supplementary file 3 — Figure S3. Posterior distributions from geographic cline analyses. [file ECE3-6-5013-s003.pdf]

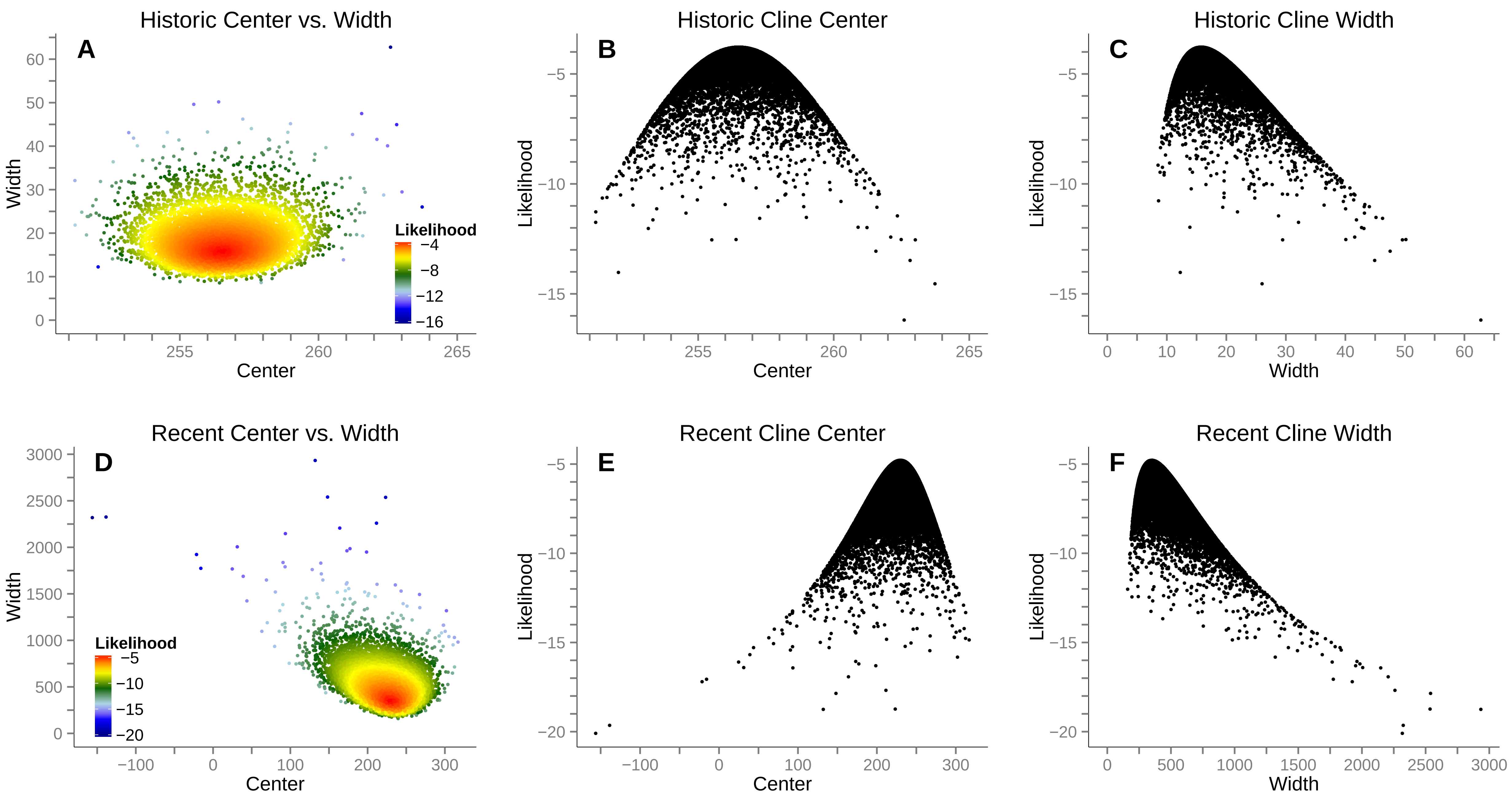
Historic Center vs. Width
A
Width
Center
Likelihood
−4
−8
−12
−16
Historic Cline Center
B
Likelihood
Center
Historic Cline Width
C
Likelihood
Width
Recent Center vs. Width
D
Width
Center
Likelihood
−5
−10
−15
−20
Recent Cline Center
E
Likelihood
Center
Recent Cline Width
F
Likelihood
Width
