# Supplementary material for: Quantifying the spatiotemporal dynamics in a chorus frog (Pseudacris) hybrid zone over 30 years
Source: Ecol Evol. 2016 Jun 26;6(14):5013–31. doi: 10.1002/ece3.2232 (PMC4979724; doi:10.1002/ece3.2232)
Supplement: Supplementary file 5 [file ECE3-6-5013-s005.docx]

**Supporting information**

**Figure S1. STRUCTURE Plots.** STRUCTURE results are shown for the historical and recent datasets. Panels show K=2, K=3, and K=4 as indicated. Each vertical line indicates one individual, and the colors represent the probability of that individual belonging to a specific genetic cluster. Populations H1 through H7 (historical) and A through P (recent) are labeled below the plots, and species designations are listed above each population.

**Figure S2. Hybrid Index Score Comparisons.** Values of hybrid index (*h)* for each putative hybrid individual are indicated by points with confidence intervals, representing the proportion of alleles inherited from either species. A value of 1 indicates pure *P. fouquettei,* and 0 indicates pure *P. nigrita*. Historical and recent populations are matched as they were for stratified subsampling analyses.

**Figure S3. Posterior Distributions from Geographic Cline Analyses.** Plots of posterior distributions from hzar analyses of historical data (A, B, C) and recent data (D, E, F) under the 2 parameter (no tails) model. A and D show cline width versus cline center, and warmer colors indicate better likelihoods. B and E show cline center versus likelihood, and C and F show cline width versus likelihood. Note that axes values differ among plots.

**Table S1. Complete Listing of Specimens**. All individuals used in this study are listed by their Field Identification number. Historical individuals have Field ID’s starting with an H for “historical,” while recent individuals have Field ID’s starting with ECM. “Test Pop” indicates which population the individual was included in for all analyses after pooling nearby recent collection sites, and “Collection Site” indicates the original designation (M number for recent data) where the individual was collected. “Species” was determined through *h*, the hybrid index score, where hybrid individuals have *h* between 0.25 and 0.75. Individuals with *h* between 0.0 and 0.25 are *P. nigrita*, and individuals with *h* between 0.75 and 1.0 are *P. fouquettei*. Latitude and longitude are given for the original collection location, prior to pooling recent populations. Individual ECM0264 (from M22) was omitted from all analyses because its distance from any other recent collection site prevented it from being pooled into a test population.

**Table S2. Microsatellite Marker Information**. MPLEX column indicates which of the four multiplexes contain each locus. MPLEX 1 used the ROX 500 size standard, and MPLEX 2-4 used the LIZ 500 size standard. Hist. Size and Recent Size give the range of the repeat length in base pairs in each dataset. “# All. Hist.” and “# All. Rec.” indicate the number of alleles found at each locus in the historical and recent dataset, respectively. Label indicates the fluorescent label that was attached to the forward primer. Asterisks by locus name indicate 4 loci that were discarded from analysis due to low amplification success (P_fer_c101070) or high levels of missing data or null alleles (A_C08d, P_fer_G79VC, P_fer_A7NK3_2).

**Table S3. FST and P-values for Combined Collection Site Pairs**. Each recent collection site (represented by M numbers) that was pooled with at least one more collection site to form recent populations is compared to each of its population counterparts, using FST as measure of genetic differentiation between each collection site. None of the comparisons were significant after a sequential Bonferroni correction.
